# Peptidoglycan Suppresses Melanoma Growth in a Murine Model with Local Immune Activation

**DOI:** 10.3390/ijms27198584

**Published:** 2026-09-25

**Authors:** Ayaka Ichikawa, Takehisa Nakanishi, Mai Nishimura, Masako Ichishi, Yoshiaki Matsushima, Makoto Kondo, Keiichi Yamanaka

**Affiliations:** 1 Department of Dermatology, Graduate School of Medicine, Mie University, Tsu 514-8507, Mie, Japan; ichika-a@med.mie-u.ac.jp (A.I.); t-nakanishi@clin.medic.mie-u.ac.jp (T.N.); ishika-m@clin.medic.mie-u.ac.jp (M.N.); matsushima-y@clin.medic.mie-u.ac.jp (Y.M.); kondomak@med.mie-u.ac.jp (M.K.); 2 Department of Oncologic Pathology, Graduate School of Medicine, Mie University, Tsu 514-8507, Mie, Japan; masako-i@doc.medic.mie-u.ac.jp

**Keywords:** melanoma, *Cutibacterium acnes*, peptidoglycan, lipoteichoic acid, innate immunity, Toll-like receptor 2, type 1-biased immune activation

## Abstract

*Cutibacterium acnes* (*C. acnes*), a Gram-positive cutaneous commensal, has been reported to have antitumor activity in melanoma, but the responsible bacterial components and innate immune pathways remain unclear. Using 8-week-old female C57BL/6NCrSlc mice bearing syngeneic B16-F10 melanoma, we examined heat-killed *Cutibacterium acnes* (*C. acnes*) and bacterial cell-wall components in an early local co-administration/booster regimen. *C. acnes* was fractionated into soluble and insoluble components, and peptidoglycan (PG) and lipoteichoic acid (LTA) were evaluated for cytokine induction in splenocytes and CD11b^+^ myeloid cells in vitro and for tumor control in vivo. Heat-killed *C. acnes* suppressed melanoma outgrowth, while the insoluble fraction retained TNF-α-inducing and antitumor activity. Both PG and LTA induced TNF-α production in vitro, whereas only PG significantly suppressed melanoma growth in vivo under the tested conditions. PG treatment was associated with extensive tumor necrosis and increased inflammatory cell infiltration. Pharmacologic inhibition of TLR2 or RIPK2 did not fully reverse PG-mediated tumor suppression. Thus, purified Gram-positive PG demonstrated local antitumor immunomodulatory activity and reproduced several features observed with the *C. acnes* insoluble fraction in this murine melanoma model.

## 1. Introduction

Melanoma is a highly immunogenic malignancy in which outcomes are strongly influenced by the quality of immune activation within the tumor microenvironment. In patients with unresectable, recurrent, or metastatic melanoma, immune checkpoint blockade, particularly anti-PD-1/PD-L1-based therapy, has improved clinical outcomes; however, many patients do not achieve durable benefit [1,2,3,4]. Therefore, strategies that enhance antitumor immunity directly at the tumor site may be useful as adjunctive approaches in advanced melanoma, rather than as stand-alone treatments for surgically resectable primary melanoma. Locally delivered immunomodulators that engage innate immune sensing and promote type 1-biased inflammation are therefore being explored to convert poorly responsive advanced tumors into immunologically active lesions [1,2,3,4,5,6,7,8].

Cutaneous commensal bacteria provide an underutilized source of such cues. *Cutibacterium acnes* (*C. acnes*), a dominant Gram-positive resident of pilosebaceous units, activates skin immunity via pattern-recognition receptors including Toll-like receptor 2 (TLR2). In acne, *C. acnes* drives production of proinflammatory and Th1-polarizing cytokines such as TNF-α and IL-12, consistent with a type 1-skewed program [9,10]. Importantly, intratumoral delivery of heat-killed *C. acnes* suppresses melanoma growth in immunocompetent mice, accompanied by granulomatous inflammation and enhanced Th1 responses [11]. However, the bacterial structures responsible for this activity and the extent to which defined innate pathways mediate tumor control remain unclear.

Gram-positive cell walls contain conserved motifs that serve as ligands for innate receptors, including peptidoglycan (PG) and lipoteichoic acid (LTA). These components can activate TLR2 and cytosolic nucleotide-binding oligomerization domain (NOD) pathways to trigger NF-κB-dependent inflammatory cytokine production [12,13,14,15]. Yet innate sensing of Gram-positive bacteria is often not explained by a single ligand–receptor pair; instead, overlapping and partially redundant pathways integrate signals from complex cell wall structures [16,17,18,19]. In tumors, particulate microbial material—such as insoluble cell wall scaffolds—may further sustain local inflammation, tissue remodeling, and antigen release, processes linked to efficient priming of adaptive antitumor immunity [20,21]. Nevertheless, few studies have directly compared defined Gram-positive cell wall constituents for antitumor efficacy in vivo.

Because PG forms the insoluble backbone of the Gram-positive wall, we hypothesized that a purified Gram-positive PG preparation could reproduce some of the immunostimulatory and antitumor features associated with the insoluble *C. acnes* fraction. We also examined LTA as another representative Gram-positive cell-wall component with known innate immune activity. Whether PG-driven effects require canonical TLR2 or NOD2/RIPK2 signaling, or instead arise from convergent innate programs within the tumor microenvironment, has not been systematically examined [22,23].

In this study, we used a syngeneic B16-F10 melanoma model to validate tumor suppression by heat-killed *C. acnes* under a defined injection–booster regimen [11,24]. This model was not designed to evaluate prophylactic vaccination before tumor challenge or treatment of established tumors, but rather to provide a controlled proof-of-concept system for identifying microbial structural components with local immunomodulatory activity. We then performed functional fractionation to distinguish soluble from insoluble components. We compared PG and LTA for myeloid activation in vitro and melanoma control in vivo, interrogated TLR2 and NOD2/RIPK2 contributions using pharmacologic inhibitors [22,23,25], and profiled treated tumors with emphasis on type 1 cytokines and T-cell infiltration [20,21]. Together, our findings support a structurally defined, microbial-derived approach to locally reprogram the melanoma microenvironment toward effective type 1-biased immune activation and provide a framework for rational development of intratumoral immunomodulatory adjuvants [26,27,28,29]. These findings support purified Gram-positive PG as a candidate structural trigger of local type 1-biased immune activation under the tested conditions.

## 2. Results

### 2.1. Heat-Killed Cutibacterium acnes Suppresses Melanoma Growth In Vivo

We first evaluated the effects of heat-killed *C. acnes* in a syngeneic B16-F10 melanoma model using the early local co-administration and booster regimen described in the Section 4. Subcutaneous co-injection of B16-F10 melanoma cells with heat-killed *C. acnes* on day 0, followed by reagent-only booster administration at the same site on day 7, resulted in marked suppression of tumor growth compared with PBS-treated controls (Figure 1A,B). Tumors in *C. acnes*-treated mice remained significantly smaller than those in PBS-treated controls during the 21-day tumor-growth observation period. In a separate cohort used for survival analysis, mice receiving heat-killed *C. acnes* showed significantly delayed endpoint progression compared with control animals during the 32-day monitoring period. Consistent with reduced tumor growth, Kaplan–Meier survival analysis demonstrated a significantly prolonged endpoint-free survival in mice receiving heat-killed *C. acnes* compared with controls (Figure 1C). Histopathological examination of tumors harvested at the experimental endpoint revealed distinct differences between treatment groups. PBS-treated tumors exhibited dense sheets of viable melanoma cells with minimal necrosis. In contrast, tumors from *C. acnes*-treated mice showed prominent inflammatory cell infiltration, extensive tumor necrosis, and granulomatous changes (Figure 1D). These findings indicate that heat-killed *C. acnes* suppresses melanoma outgrowth under this early local regimen and is associated with tumor necrosis and inflammatory cell infiltration. To test whether the tumor-suppressive effect of heat-killed *C. acnes* could be explained by a direct effect on melanoma cells, we cultured B16-F10 cells with PBS or heat-killed *C. acnes* (33 µg/well) under immune-cell-free conditions. Heat-killed *C. acnes* did not significantly reduce the number of recovered viable B16-F10 cells after 24 h compared with the PBS control (Figure 1E). These findings suggest that marked direct cytotoxicity toward B16-F10 cells is unlikely to fully account for the antitumor effect observed in vivo under the tested conditions.

### 2.2. The Operationally Defined Insoluble Fraction of C. acnes Retains TNF-α-Inducing and Antitumor Activity

To identify the bacterial components responsible for the antitumor activity of heat-killed *C. acnes*, we fractionated bacterial preparations into soluble and insoluble fractions and assessed their immunostimulatory and antitumor properties (Figure 2A). When applied to murine splenocyte cultures, the insoluble fraction induced robust TNF-α production, whereas the soluble fraction elicited minimal cytokine responses under equivalent conditions (Figure 2B). We next evaluated whether this differential immunostimulatory activity translated into antitumor efficacy in vivo. Subcutaneous co-injection of B16-F10 melanoma cells with the insoluble fraction, followed by a booster administration at the same site, resulted in significant suppression of tumor growth compared with PBS-treated controls (Figure 2C). Tumor volumes remained consistently reduced throughout the observation period, recapitulating the antitumor phenotype observed with unfractionated heat-killed *C. acnes*. Together, these findings demonstrate that the operationally defined insoluble fraction of *C. acnes* retains both TNF-α-inducing activity in vitro and antitumor efficacy in vivo.

### 2.3. Peptidoglycan and Lipoteichoic Acid Activate TNF-α Production in Splenocytes and Splenic CD11b^+^ Myeloid Cells

To test whether representative Gram-positive cell-wall components could reproduce the immunostimulatory activity associated with the insoluble fraction of *C. acnes*, we next evaluated the capacity of commercially available purified PG and LTA preparations to induce inflammatory cytokine production in vitro. Stimulation of murine splenocytes with either PG or LTA resulted in robust TNF-α secretion compared with unstimulated controls, whereas the PG monomeric components N-acetylmuramic acid (NAM) and N-acetylglucosamine (NAG) failed to elicit comparable responses (Figure 3A).

To assess the contribution of innate immune sensing pathways, splenocytes were pretreated with pharmacologic inhibitors targeting TLR2 or NOD2/RIPK2 signaling prior to stimulation. Inhibition of TLR2 signaling with C29 showed the most consistent inhibitory effect, whereas CU-CPT22 and WEHI-345 produced partial effects depending on the stimulus and cell population (Figure 3B). Because myeloid cells can contribute to inflammatory cytokine production following microbial stimulation, we next examined responses in MACS-purified splenic CD11b^+^ cells as an enriched myeloid-cell population. Consistent with results obtained using total splenocytes, PG and LTA induced high levels of TNF-α production in CD11b^+^ cells. C29 showed the most consistent inhibitory effect on PG- and LTA-induced TNF-α production, whereas CU-CPT22 and WEHI-345 produced partial effects depending on the stimulus and cell population (Figure 3C). Together, these findings indicate that both PG and LTA induced TNF-α production in splenocytes and splenic CD11b^+^ myeloid cells under the tested conditions.

### 2.4. Peptidoglycan, but Not Lipoteichoic Acid, Suppresses Melanoma Outgrowth Under the Early Local Regimen

Given that both PG and LTA robustly induced TNF-α production in vitro, we next compared their effects on melanoma outgrowth under the same early local co-administration and booster regimen. Mice were subcutaneously inoculated with melanoma cells, together with PG or LTA, on day 0, followed by a booster injection at the same site on day 7. The total administered doses were 2 mg/mouse for PG and 2.5 mg/mouse for LTA, divided equally between the day 0 and day 7 injections. Under this regimen, PG treatment resulted in a significant reduction in tumor growth compared with PBS-treated controls, whereas LTA failed to confer a comparable level of tumor suppression (Figure 4A). Histopathological analysis of harvested tumors revealed marked differences between the treatment groups. Tumors from PG-treated mice exhibited extensive necrosis accompanied by granulomatous inflammation and tissue remodeling, whereas LTA-treated tumors showed prominent inflammatory cell infiltration but largely preserved viable tumor architecture (Figure 4B). To assess whether PG-mediated tumor suppression was associated with adaptive immune activation, we performed flow cytometry to analyze tumor-infiltrating immune cells. Flow cytometric analysis of pooled tumor-infiltrating cells suggested that PG treatment increased the frequencies of IFN-γ- and TNF-α-producing monocyte/myeloid cells, CD4^+^ T cells, and CD8^+^ T cells compared with PBS-treated controls (Figure 4C). In contrast, LTA treatment induced only modest changes in these populations.

### 2.5. PG-Mediated Tumor Suppression Is Not Fully Reversed by Pharmacologic Co-Administration of TLR2 or RIPK2 Inhibitors

To examine whether canonical innate immune sensing pathways contributed to PG-associated tumor suppression under this early local regimen, we evaluated the impact of pharmacologic inhibition of TLR2 and NOD2/RIPK2 signaling. B16-F10 melanoma cells were co-injected with PG on day 0, followed by a booster administration on day 7. TLR2 inhibitors C29, CU-CPT22, and/or the RIPK2 inhibitor WEHI-345 were administered according to the same schedule as PG and continued throughout the observation period. Representative gross images at day 21 were consistent with the endpoint tumor-volume measurements, showing reduced tumor burden in PG-treated groups compared with PBS-treated controls (Figure 5A,B). Co-administration of TLR2 and/or RIPK2 inhibitors did not clearly abolish the PG-associated reduction in tumor burden under the tested conditions (Figure 5A,B). Individual data points are shown to illustrate inter-mouse variability in endpoint tumor volume. Histopathological analysis corroborated these findings. PBS-treated tumors consisted predominantly of viable melanoma cells with minimal necrosis, whereas tumors from PG-treated mice exhibited extensive necrosis and inflammatory cell infiltration. Similar necrosis-associated inflammatory changes, including tumor necrosis and inflammatory cell infiltration, were observed in tumors from mice receiving PG in combination with TLR2 and/or RIPK2 inhibitors, indicating that PG-associated tissue remodeling was not clearly abolished under these pharmacologic co-administration conditions (Figure 5C).

To assess systemic immune activation, TNF-α and IFN-γ levels were measured by ELISA at the experimental endpoint. No significant differences in circulating cytokine levels were detected among PG-treated groups, irrespective of inhibitor co-administration (Figure 5D), suggesting that PG-mediated tumor control is not reflected by sustained systemic cytokine elevations at this time point. Because these ELISA measurements were performed using bulk samples, they did not identify the cellular sources of TNF-α or IFN-γ. Finally, we examined the expression of inflammatory and innate immune-related genes within tumor tissues by quantitative real-time PCR. While transcripts for *Ifng*, *Nod2*, and *Ifnb1* did not differ significantly among PG-based treatment groups, expression of *Il10*, *Stat1*, and *Tmem173* was increased in subsets of inhibitor-treated tumors (Figure 5E). Collectively, these results indicate that PG-mediated melanoma suppression was not fully reversed by pharmacologic co-administration of TLR2 or RIPK2 inhibitors under the conditions tested.

## 3. Discussion

In this study, we performed functional fractionation of heat-killed *C. acnes* and found that the operationally defined insoluble fraction retained TNF-α-inducing and melanoma-suppressive activity. We subsequently evaluated representative purified Gram-positive cell-wall components and found that PG exhibited local antitumor activity in the B16-F10 melanoma model, accompanied by tumor necrosis and inflammatory changes. Because the insoluble *C. acnes* fraction was not chemically characterized and the PG preparation used in this study was not isolated from *C. acnes*, these findings do not identify PG as the component responsible for the antitumor activity of *C. acnes*. Rather, they demonstrate that purified Gram-positive PG can reproduce several functional features observed with the insoluble *C. acnes* fraction under the tested conditions. *C. acnes* is a dominant cutaneous commensal bacterium with well-established immunostimulatory properties in acne, where it activates innate immune pathways and promotes Th1-skewed cytokine responses through receptors such as TLR2 [9,10]. Building on prior observations that intratumoral administration of heat-killed *C. acnes* suppresses melanoma growth and enhances Th1 immunity in vivo [11], our findings show that purified Gram-positive PG can reproduce several immunostimulatory and antitumor features associated with the insoluble *C. acnes* fraction. However, because the insoluble fraction was not chemically characterized and the PG preparation was not derived from *C. acnes*, these findings do not identify PG as the component responsible for the antitumor activity of *C. acnes*.

Using a syngeneic B16-F10 melanoma model, we first confirmed that heat-killed *C. acnes* suppressed tumor growth and prolonged endpoint-free survival under the injection–booster regimen (Figure 1). In an immune-cell-free culture assay, heat-killed *C. acnes* did not significantly reduce viable B16-F10 cell recovery after 24 h. These findings suggest that marked direct cytotoxicity is unlikely to fully account for the antitumor effect observed in vivo, although more subtle effects on tumor-cell behavior cannot be excluded. Fractionation of *C. acnes* further showed that TNF-α-inducing and antitumor activities were retained in the operationally defined insoluble fraction (Figure 2). Because this fraction was obtained as the washed pellet after sonication and centrifugation, it was considered enriched in insoluble cell wall-associated material; however, its precise molecular composition was not determined. These findings provided the rationale for evaluating defined Gram-positive cell-wall components, including PG and LTA. Because we did not evaluate the soluble fraction in vivo, we cannot exclude the possibility that soluble *C. acnes*-derived components also exert antitumor effects through TNF-α-independent mechanisms.

We therefore focused on two representative Gram-positive cell wall components—PG and LTA—both of which are known to activate innate immune responses through pattern-recognition receptors [13,14,15]. The PG and LTA preparations used here were commercially available *Bacillus subtilis*-derived reagents and were not isolated from *C. acnes*-Mie1. They were therefore used as representative Gram-positive cell-wall preparations for functional comparison and should not be regarded as structurally equivalent to *C. acnes*-derived PG or LTA. In vitro, both PG and LTA robustly induced TNF-α production by splenocytes and purified CD11b^+^ myeloid cells, whereas the PG monomers N-acetylglucosamine and N-acetylmuramic acid lacked comparable activity (Figure 3). Although NAM and NAG are structural components of the PG backbone, neither monomer induced TNF-α production comparable to intact PG, indicating that the intact PG preparation elicited stronger innate immune activation than its individual sugar constituents. Pharmacologic inhibition was consistent with a contribution of TLR2-associated signaling to these cytokine responses, whereas inhibition of RIPK2 produced more variable effects. This pattern is consistent with previous reports suggesting that innate immune responses to Gram-positive bacterial cell-wall components reflect integrated sensing through multiple pathways rather than a single receptor–ligand interaction [15,16,17,18,19].

Despite similar cytokine-inducing capacity in vitro, PG and LTA showed different outcomes in vivo at the exploratory doses tested. PG, but not LTA, suppressed melanoma growth, induced extensive tumor necrosis under these conditions, while descriptive analysis of pooled tumor-infiltrating cells suggested higher frequencies of IFN-γ- and TNF-α-producing monocyte/myeloid cells, CD4^+^ T cells, and CD8^+^ T cells after PG treatment (Figure 4). Because tumor samples were pooled within each treatment group, these flow cytometry findings should be considered descriptive and do not permit statistical comparison among individual biological replicates. These results highlight a critical distinction between acute cytokine induction in vitro and productive antitumor immunity in vivo. LTA can exert context-dependent inflammatory or tolerogenic effects depending on bacterial origin, structure, dose, and cellular context [30]. Thus, its ability to induce TNF-α acutely in vitro does not necessarily predict antitumor activity in vivo. Consistent with this distinction, LTA induced inflammatory infiltration but did not show comparable tumor necrosis or growth suppression at the dose tested. This qualitative difference raises the possibility that structural properties of PG—such as its particulate nature, persistence within tissues, or capacity to promote the release of damage-associated molecular patterns (DAMPs)—may contribute to the conversion of innate immune activation into adaptive antitumor responses. However, these mechanisms were not directly tested in the present study and should be regarded as hypotheses for future investigation. The PG and LTA doses used in vivo were exploratory and were not selected to provide dose-equivalent or equimolar comparisons. Therefore, the present experiment does not establish a difference in the intrinsic antitumor potency of PG and LTA. Previous studies have reported antitumor activity of peptidoglycan- or teichoic acid-related bacterial components in murine tumor models [31,32]. Direct quantitative comparison across these studies is limited by differences in reagent origin, dose, and administration route.

Pharmacologic co-administration of TLR2 and/or RIPK2 inhibitors did not fully reverse PG-mediated tumor suppression or the associated necrosis and inflammatory infiltration (Figure 5). The present experiments cannot distinguish incomplete pharmacologic blockade from contributions of additional innate signaling pathways and do not establish that TLR2 or RIPK2 is dispensable for the antitumor effect of PG. In addition, bulk ELISA and qPCR did not resolve the cellular sources of TNF-α and IFN-γ, and the pooled flow cytometry experiment was descriptive. Non-pooled, higher-dimensional immune profiling will be required to define the relevant cytokine-producing populations and T-cell functional states.

One possible explanation for the incomplete effect of TLR2/RIPK2 inhibition is engagement of additional innate sensing pathways. PG can interact with peptidoglycan recognition proteins (PGRPs), which modulate innate immune activation and bacterial clearance [33]. Moreover, tumor necrosis may release host-derived nucleic acids capable of activating pathways such as cGAS–STING and type I interferon signaling [34,35,36,37]. Although PG itself is not a direct STING agonist, such secondary signaling could be consistent with the increased *Stat1* and *Tmem173* expression observed in subsets of inhibitor-treated tumors. Because these analyses were performed using bulk tumor tissue, however, the transcriptional changes cannot be assigned to specific cell populations without additional cell-resolved analyses [38].

An important limitation of this study is that treatment was initiated at the time of tumor inoculation. Thus, the model evaluates modulation of tumor initiation and early outgrowth rather than treatment of established melanoma. In addition, large B16 tumors can develop extensive spontaneous necrosis, complicating interpretation of late-stage immune and histological changes [24]. We therefore analyzed tumor-infiltrating cells at day 14 in the PG/LTA comparison, whereas later time points were used primarily for tumor-growth and endpoint analyses. Future studies in established tumor models will be required to evaluate therapeutic efficacy.

From a translational perspective, our findings support further evaluation of defined microbial-derived components as local immunomodulatory agents. Clinical precedent exists for locally delivered immune stimulation, including T-VEC and intratumoral TLR9 agonists in melanoma [27,28], while the PG derivative muramyl tripeptide has demonstrated therapeutic activity in osteosarcoma [26]. PG-based local immunomodulation may therefore warrant evaluation as an adjunct to immune checkpoint blockade, although its efficacy must first be established in models of established melanoma. The present findings provide a rationale for such studies by showing that purified Gram-positive PG can reproduce several immunostimulatory and antitumor features associated with the insoluble *C. acnes* fraction.

Finally, our findings may be viewed in the broader context of host–microbiome interactions in cancer immunotherapy. Commensal bacteria are increasingly recognized to influence responses to chemotherapy and immune checkpoint blockade through modulation of antitumor immunity [39,40,41,42,43,44,45,46]. Our results extend this concept by suggesting that a defined bacterial cell-wall component can itself contribute to local antitumor immune activation, providing a rationale for further investigation of microbial-derived immunomodulatory strategies [16,21,47].

## 4. Materials and Methods

### 4.1. Mice

Female wild-type (WT) C57BL/6NCrSlc mice (RRID: IMSR_SLC:002) were purchased from Japan SLC, Inc. (Shizuoka, Japan) and maintained under specific pathogen-free conditions. All animal experiments were approved by the Animal Care and Use Committee of Mie University Graduate School of Medicine (protocol no. 21-27-7; approved 24 May 2024) and were conducted in accordance with institutional guidelines and national regulations. Mice were housed under controlled environmental conditions (temperature 21 ± 2 °C; humidity 60%; 12-h light/12-h dark cycle) with ad libitum access to food and water. Unless otherwise indicated, experiments were performed using 8-week-old mice, which were sequentially allocated to experimental groups prior to tumor inoculation or treatment. No formal randomization was performed. All experiments were performed with *n* = 5 per group, except for Figure 1B, which was performed with *n* = 6 per group, and a total of 97 mice were used in this study. We did not establish prespecified inclusion or exclusion criteria, and we excluded no animals or data points from the analyses.

### 4.2. Melanoma Cells and Tumor Inoculation

We obtained the murine melanoma cell line B16-F10 from the American Type Culture Collection (ATCC; Manassas, VA, USA) and cultured it in RPMI-1640 medium (Sigma-Aldrich, St. Louis, MO, USA) supplemented with 10% fetal bovine serum (HyClone Laboratories, South Logan, UT, USA). Cells were maintained at 37 °C in a humidified incubator with 5% CO_2_ and were routinely passaged to ensure logarithmic growth. Although the supplier commonly recommends DMEM for B16 cells, we used RPMI-1640 in this study to align with our established laboratory protocol and the immune-cell culture system used for the in vitro stimulation assays. We used cells after limited passage and prepared all experimental groups under the same culture conditions to allow internal comparison among treatment groups. For tumor inoculation, B16-F10 melanoma cells were harvested, washed, and resuspended in sterile phosphate-buffered saline (PBS). We mixed 1.0 × 10^6^ viable cells with the indicated test reagents immediately before injection and administered them subcutaneously into the dorsal midline skin, slightly cranial to the pelvis, of 8-week-old female WT C57BL/6NCrSlc mice using a single syringe. We used the same anatomical region for all tumor inoculations. Cell viability was confirmed to be greater than 95% by trypan blue exclusion prior to inoculation. Unless otherwise specified, tumor-bearing mice received treatments according to an early local co-administration and reagent-only booster schedule, in which B16-F10 melanoma cells were inoculated only once on day 0 together with the indicated test reagents, and the corresponding reagents alone were re-administered at the same site on day 7 without additional melanoma cells.

### 4.3. C. acnes Preparation

*C. acnes* was isolated from acne lesions of human donors and cultured under anaerobic conditions in brain–heart infusion medium (BD BBL™, Becton, Dickinson and Company, Sparks, MD, USA; Cat. No. 211059) supplemented with L-cysteine (Wako Pure Chemical Industries, Ltd., Osaka, Japan) and Tween 80 (Tokyo Chemical Industry Co., Ltd., Tokyo, Japan; Cat. No. T0546), as previously described [48]. Multiple single-colony isolates were expanded, heat-killed, and screened for cytokine-inducing activity in immune-cell stimulation assays by measuring TNF-α in culture supernatants by ELISA (R&D Systems, Minneapolis, MN, USA). The isolate that reproducibly showed high cytokine-inducing activity was selected for subsequent experiments and designated *C. acnes-Mie1* [48]. The use of human-derived bacterial isolates and associated clinical samples was approved by the Institutional Review Board of Mie University (approval no. 575; 3 October 2005), in accordance with institutional guidelines and the Declaration of Helsinki. 

Bacterial cultures were harvested, washed extensively with sterile PBS, and heat-killed at 60 °C for 60 min to ensure complete loss of viability. Heat-killed bacteria were then lyophilized and stored at −80 °C until use. Before use, lyophilized preparations were reconstituted in sterile PBS and administered at 3.3 mg/mouse in the heat-killed *C. acnes* experiment, as specified in the corresponding figure legend [11]. To prepare *C. acnes*-derived soluble and insoluble fractions, we collected cultured *C. acnes*-Mie1 by centrifugation at 4000× *g* for 10 min and heat-killed it at 60 °C for 60 min. The heat-killed bacterial pellet was resuspended in 10 mL sterile PBS and disrupted by sonication on ice for five 30-s cycles. The disrupted suspension was passed through a 70-µm cell strainer to remove large aggregates and then centrifuged at 12,000× *g* for 20 min at 4 °C. The supernatant was collected as the soluble fraction, whereas the pellet was collected as the insoluble fraction. The insoluble fraction was washed two to three times with sterile PBS under the same centrifugation conditions and resuspended in sterile PBS. For in vitro stimulation assays, the insoluble fraction was added at 6.25 µg/well, whereas the soluble fraction was added at the same volume. For in vivo injection experiments, the insoluble fraction was administered at 3.3 mg/mouse on day 0, followed by re-administration of the same dose at the same site on day 7. Aliquots were stored at −80 °C until use and mixed thoroughly before application. The soluble and insoluble fractions were operationally defined based on this fractionation procedure and were not chemically homogeneous purified preparations.

### 4.4. In Vitro Assessment of the Direct Effect of Heat-Killed C. acnes on B16-F10 Cells

B16-F10 cells were seeded in 24-well plates at 1.0 × 10^4^ cells/well in 500 µL of complete RPMI-1640 medium and allowed to adhere overnight. The following day, the medium was replaced with 500 µL of fresh complete RPMI-1640 medium containing either PBS vehicle or heat-killed *C. acnes* at 33 µg/well, with the same vehicle volume used in both groups. Cells were incubated for 24 h at 37 °C in 5% CO_2_. The 33 µg/well dose was selected by scaling the ratio of heat-killed *C. acnes* mass to the initial B16-F10 cell number used in the in vivo experiment, in which 3.3 mg of heat-killed *C. acnes* was co-administered with 1.0 × 10^6^ B16-F10 cells. This calculation was used as a pragmatic basis for dose selection and was not intended to reproduce the local tissue concentration in vivo. After incubation, floating cells in the culture supernatant and adherent cells detached by trypsinization were pooled. The cell suspension was mixed 1:1 with 0.4% (*w*/*v*) Trypan Blue Solution (FUJIFILM Wako Pure Chemical Corporation, Osaka, Japan; Cat. No. 207-17081), and viable cells were manually counted using a hemocytometer.

### 4.5. Splenocyte and CD11b^+^ Cells Preparation

Spleens were harvested from euthanized mice and mechanically dissociated through a 70-µm cell strainer to generate single-cell suspensions. Red blood cells were removed using ammonium–chloride–potassium (ACK) lysis buffer (Thermo Fisher Scientific, Waltham, MA, USA), followed by washing with PBS. Splenocytes were resuspended in RPMI-1640 medium supplemented with 10% fetal bovine serum and used immediately for in vitro stimulation assays.

For isolation of CD11b^+^ myeloid cells, splenocyte suspensions were subjected to magnetic-activated cell sorting (MACS) using anti-CD11b microbeads (Miltenyi Biotec, Bergisch Gladbach, Germany, Cat. No. 130-049-601), according to the manufacturer’s instructions. The purity of isolated CD11b^+^ cells routinely exceeded 95%, as assessed by flow cytometry. Purified cells were washed and resuspended in complete RPMI-1640 medium for downstream functional assays.

### 4.6. In Vitro Stimulation of Splenocytes and CD11b^+^ Cells with PG/LTA and Inhibitor Pretreatment

Total splenocytes were used as an initial mixed immune-cell screening system to assess the cytokine-inducing activity of PG and LTA in a heterogeneous immune-cell context. MACS-purified splenic CD11b^+^ cells were then used to enrich for myeloid cells and to determine whether the TNF-α response was retained in the splenic myeloid compartment; these experiments were not intended to define macrophage-specific responses.

Splenocytes or MACS-purified CD11b^+^ cells were plated in 96-well culture plates at 0.6 × 10^6^ cells/well in 150 µL of complete RPMI-1640 medium. Cells were stimulated with peptidoglycan (PG; Sigma-Aldrich, St. Louis, MO, USA, Cat. No. 69554), lipoteichoic acid (LTA; Sigma-Aldrich, St. Louis, MO, USA, Cat. No. L3265), N-acetyl-D-glucosamine (NAG; Sigma-Aldrich, St. Louis, MO, USA, Cat. No. A3286), or N-acetylmuramic acid (NAM; Sigma-Aldrich, St. Louis, MO, USA, Cat. No. A3007) at 10 µg/well, for 3 h at 37 °C. PG and LTA concentrations were selected with reference to previous in vitro studies using these bacterial cell-wall components for immune-cell stimulation [49,50,51]. These PG and LTA preparations were commercially available purified reagents derived from *Bacillus subtilis* and were not isolated directly from *C. acnes*-Mie1. NAG and NAM were included at the same mass dose as structural sugar controls for PG rather than as established innate immune agonists [47]. 

For inhibitor studies, cells were pretreated for 3 h with the TLR2 inhibitors TLR2-IN-C29 (C29; CAS 363600-92-4; Selleck Chemicals, Houston, TX, USA, Cat. No. S6597) or CU-CPT22 (CAS 1416324-85-0; Selleck Chemicals, Houston, TX, USA, Cat. No. S8677), or with the RIPK2 inhibitor WEHI-345 (CAS 1354825-58-3; MedChemExpress, Monmouth Junction, NJ, USA, Cat. No. HY-18937), prior to stimulation with PG or LTA. C29, CU-CPT22, and WEHI-345 were dissolved in dimethyl sulfoxide (DMSO) at 100 mg/mL. For inhibitor studies, cells were pretreated for 3 h with C29 (150 µM), CU-CPT22 (150 µM), or WEHI-345 (150 µM) prior to stimulation with PG or LTA. Vehicle control groups received an equivalent dilution of DMSO in PBS according to the same schedule. Control wells received an equivalent concentration of vehicle (dimethyl sulfoxide, DMSO; CultureSure, FUJIFILM Wako Pure Chemical Corporation, Osaka, Japan, Cat. No. 031-24051). Following stimulation, culture supernatants were collected, and TNF-α concentrations were quantified by enzyme-linked immunosorbent assay (ELISA) according to the manufacturer’s instructions.

### 4.7. Early Local Co-Administration and Booster Treatment Schedule

Mice were sequentially allocated to experimental groups prior to tumor inoculation. No formal measures were taken to control treatment order or cage position; however, strain, sex, age, and housing conditions were standardized across groups. Unless otherwise specified, all in vivo experiments were conducted using a two-dose injection-booster regimen. This regimen was designed as a proof-of-concept model to test whether microbial components could modulate the local immune environment during tumor initiation and early outgrowth. It was not intended to represent prophylactic vaccination before tumor inoculation or therapeutic treatment of established tumors. On day 0, B16-F10 melanoma cells were injected subcutaneously into the dorsal midline skin, slightly cranial to the pelvis, together with the indicated test reagents at the same injection site. On day 7, the corresponding reagents were re-administered at the identical site as a booster injection in the absence of tumor cells. The test reagents administered on days 0 and 7 included heat-killed *C. acnes*, the insoluble cell wall-associated fraction of *C. acnes*, PG, or LTA, as indicated in each experiment. Unless otherwise stated, the total administered dose per mouse was 2 mg for PG and 2.5 mg for LTA, divided equally between the day 0 co-administration and the day 7 reagent-only booster injection. These doses were used to compare the antitumor effects of PG and LTA under the tested local administration regimen, but were not designed to determine dose-normalized or equimolar relative potency between the two reagents. The dose of PG used in the present study was within the range of previous in vivo studies using B16-F10 melanoma-bearing mice, in which peptidoglycan monomer was administered at approximately 1 mg per mouse and showed antimetastatic activity [31]. For LTA, no directly comparable local administration regimen using a *Bacillus subtilis*-derived preparation was identified. Therefore, the LTA dose was selected as an exploratory local dose for comparison with PG under the tested conditions.

For pathway inhibition studies, pharmacologic inhibitors were administered three times per week until day 21 after tumor inoculation. Because no directly comparable subcutaneous dosing regimen was available, inhibitor dosing was selected with reference to previous in vivo CU-CPT22 administration in mice, in which CU-CPT22 was administered intraperitoneally at 3 mg/kg every two days [52,53]. The final dose was selected as a feasible local dose that balanced inhibitor solubility and minimized DMSO exposure. C29, CU-CPT22, and WEHI-345 were dissolved in DMSO at 100 mg/mL and diluted in warmed sterile PBS immediately before administration. Mice received approximately 110 µg inhibitor per injection in 200 µL. Vehicle-control mice received an equivalent DMSO dilution in PBS on the same schedule. Tumor size was measured on days 7, 14, and 21, and tumors were harvested on day 21 for downstream analyses.

In the other in vivo experiments, tumor growth was monitored for the periods indicated in the corresponding figure legends. In the heat-killed *C. acnes* experiment, survival and tissue-collection experiments were performed using separate cohorts of mice. Endpoint-free survival was monitored until day 32 after tumor inoculation in a separate cohort and was defined according to predefined humane endpoint criteria based on tumor burden and general condition, rather than spontaneous death. Tumor tissues for histological and immunological analyses were harvested on day 21 in a separate cohort before excessive tumor burden developed in control melanoma-bearing mice.

### 4.8. Tumor Monitoring and Histological Study

Tumor growth was monitored at regular intervals following tumor inoculation. Tumor dimensions were measured using digital calipers, and the longest diameter (length, L) and the perpendicular shorter diameter (width, W) were recorded. Tumor volume was calculated using the ellipsoid formula: 0.52 × L × W^2^ [54,55]. Measurements were performed in a blinded manner whenever feasible. For histological analysis, tumor tissues were harvested at the indicated time points, fixed in 10% neutral-buffered formalin, embedded in paraffin, and sectioned. Sections were stained with hematoxylin and eosin (H&E) according to standard protocols. Histopathological evaluation was performed to assess tumor architecture, inflammatory cell infiltration, and the presence and extent of tumor necrosis. Representative images were acquired using a light microscope under the indicated magnifications.

### 4.9. Tissue Sampling

Mice were euthanized by carbon dioxide inhalation at the indicated time points. Tumor tissues and spleens were harvested immediately after euthanasia. Tumors were divided for downstream analyses, with portions fixed in formalin for histological evaluation and the remaining tissue either snap-frozen in liquid nitrogen or processed fresh for cell isolation and molecular analyses, as specified for each experiment.

### 4.10. Cell Isolation from Spleen and Tumor

Single-cell suspensions from spleens were prepared by mechanical dissociation through a 70-µm cell strainer, followed by red blood cell lysis using ammonium–chloride–potassium (ACK) lysis buffer. Cells were washed with PBS and resuspended in RPMI-1640 medium supplemented with 10% fetal bovine serum for downstream analyses. For isolation of tumor-infiltrating cells, excised tumor tissues were minced into small fragments using sterile scissors and mechanically dissociated. Tissue suspensions were filtered through a 70-µm cell strainer to remove debris, followed by density gradient centrifugation using Histopaque-1083 (Sigma-Aldrich, St. Louis, MO, USA; Cat. No. 10831-100ML) to enrich mononuclear cells. Isolated cells were washed and resuspended in complete RPMI-1640 medium for subsequent flow cytometric and functional analyses.

### 4.11. Flow Cytometry

Tumor-infiltrating leukocytes were prepared from tumor-bearing dorsal skin tissues as follows. Tumor tissues from PBS-, PG-, and LTA-treated mice were excised on day 14 after tumor inoculation. For each treatment group, tumor tissues from five mice were pooled before tissue dissociation, cell culture, staining, and flow cytometric acquisition. Therefore, the flow cytometry data shown in Figure 4C were analyzed descriptively and were not subjected to statistical comparison across biological replicates. Tissues were minced into small fragments and mechanically dissociated using an electric homogenizer. Samples were digested in RPMI 1640 containing 1% fetal calf serum, LiberaseTM (85 μg/mL; Roche, Basel, Switzerland), and DNase I (0.01%; Roche) for 60 min at 37 °C with vigorous agitation. Cell suspensions were sequentially filtered through 100-μm and 30-μm nylon meshes and centrifuged at 1200× *g* for 7 min. Red blood cells were lysed using ACK lysis buffer, followed by washing with PBS. Debris was removed using Debris Removal Solution (Miltenyi Biotec, Auburn, CA, USA) according to the manufacturer’s instructions. Mononuclear cells were further purified by density gradient centrifugation and resuspended in cold staining buffer. For intracellular cytokine analysis, cells were stimulated ex vivo with phorbol 12-myristate 13-acetate (PMA; Funakoshi Co., Ltd., Tokyo, Japan; 25 ng/mL) and ionomycin (FUJIFILM Wako Pure Chemical Corporation, Osaka, Japan; 1 μg/mL) in the presence of brefeldin A (BioLegend, San Diego, CA, USA; 5 μg/mL) for 4 h at 37 °C in 5% CO_2_. Dead cells were excluded using LIVE/DEAD™ Fixable Aqua Dead Cell Stain Kit (Thermo Fisher Scientific, Waltham, MA, USA). Fc receptors were blocked with anti-mouse CD16/32 antibody (clone 93, BioLegend, Cat#101302, RRID: AB_312801) prior to surface staining. Surface staining was performed at 4 °C for 30 min using fluorochrome-conjugated antibodies against PE-Cy7 anti-mouse CD3 (clone 145-2C11, BD Biosciences, Franklin Lakes, NJ, USA, Cat#557654, RRID: AB_396769), BV421 anti-mouse CD4 (clone GK1.5, BioLegend, Cat#100443, RRID: AB_11218833), APC-Cy7 anti-mouse CD8a (clone 53-6.7, BD Biosciences, Cat#557654, RRID: AB_396773), PE anti-mouse CD14 (clone Sa14-2, BioLegend, Cat#123309, RRID: AB_893245). Cells were then fixed and permeabilized using Cytofix/Cytoperm™ solution (BD Biosciences) according to the manufacturer’s protocol. Intracellular staining was performed using FITC anti-mouse TNF-α (clone MP6-XT22, BioLegend, Cat#506304, RRID: AB_315432) and PerCP-Cy5.5 anti-mouse IFN-γ (clone XMG1.2, BioLegend, Cat#505821, RRID: AB_961360). Following washing, cells were resuspended in FACS buffer (PBS containing 1% bovine serum albumin and DNase I at 50 μg/mL), filtered through 30-μm nylon mesh filter-cap tubes, and analyzed on a BD FACSLyric™ flow cytometer (BD Biosciences). Data were analyzed using FlowJo software (v10.10.0, BD Biosciences, RRID:SCR_008520). Flow cytometry data were analyzed using a sequential gating strategy. After exclusion of debris based on forward scatter and side scatter profiles, doublets were excluded using FSC-A/FSC-H parameters. Viable single cells were identified after exclusion of LIVE/DEAD Aqua-positive cells. Among viable single cells, T cells were identified as CD3^+^ cells and further separated into CD4^+^ and CD8^+^ subsets. CD14^+^ cells were analyzed as monocyte/myeloid populations. Intracellular TNF-α and IFN-γ expression was then evaluated within each gated population. The same gating strategy was applied consistently to all samples.

### 4.12. Real-Time Polymerase Chain Reaction (Real-Time PCR)

Total RNA was extracted from tumor tissues using the RNeasy Plus Mini Kit (QIAGEN, Hilden, Germany, Cat. No. 74134) according to the manufacturer’s instructions. RNA concentration and purity were assessed using a NanoDrop Lite spectrophotometer (Thermo Fisher Scientific, Waltham, MA, USA). Approximately 1 μg of total RNA was converted to cDNA using a High-Capacity RNA-to-cDNA Kit (Applied Biosystems, Waltham, MA, USA). The TaqMan Universal PCR Master Mix II with UNG (Applied Biosystems) was used to measure the mRNA expression of *Tnf* (Mm00443258_m1), *Ifng* (Mm01168134_m1), *Il10* (Mm99999062_m1), *Ifnb1* (Mm00439552_s1), *Nod2* (Mm00467543_m1), *Tmem173*(Mm01158117_m1), and *Stat1* (Mm00439531_m1). Glyceraldehyde-3-phosphate dehydrogenase (*Gapdh*; Mm99999915_g1) was used as an internal control. Commercially available TaqMan Gene Expression Assays were purchased from Thermo Fisher Scientific. The amplification was performed in a LightCycler 96 System (Roche Diagnostics, Indianapolis, IN, USA). The cycling parameters were as follows: 50 °C for 120 s, 95 °C for 600 s, followed by 40 cycles of amplification at 95 °C for 15 s, and 60 °C for 60 s. Relative transcript abundance was calculated using a ΔCt-based method as 2^−ΔCt^, where ΔCt = Ct_target gene − Ct_*Gapdh*. Data are presented as gene/*Gapdh* expression ratios. Because commercially available TaqMan Gene Expression Assays were used, primer and probe sequences are proprietary and are therefore not listed individually.

### 4.13. ELISA

Cytokine concentrations in culture supernatants, tumor homogenates, or spleen homogenates were quantified using commercially available ELISA kits for TNF-α and IFN-γ (R&D Systems, Minneapolis, MN, USA), according to the manufacturer’s instructions. Samples exceeding the upper limit of quantification were diluted appropriately to ensure measurements within the linear range of the assay. Absorbance values were measured using a Multiskan FC microplate reader (Thermo Fisher Scientific, Waltham, MA, USA). Cytokine concentrations were calculated from standard curves generated with recombinant cytokine standards provided with each kit.

### 4.14. Statistical Analysis

Statistical analyses were performed using Prism software version 10 (GraphPad, San Diego, CA, USA, RRID:SCR_002798). Data are presented as mean ± standard error of the mean (SEM) unless otherwise indicated. For comparisons between two groups, the Mann–Whitney U test was used. For nonparametric comparisons among three or more groups, the Kruskal–Wallis test followed by Dunn’s multiple-comparisons test was applied. For parametric comparisons, one-way ANOVA followed by Tukey’s multiple-comparisons test was used. For longitudinal tumor growth analyses, group differences were assessed at prespecified time points, as indicated in the corresponding figure legends, using the Mann–Whitney U test. Survival curves were generated using the Kaplan–Meier method and compared using the log-rank (Mantel–Cox) test as the primary analysis, with the Gehan–Breslow–Wilcoxon test used as a supportive analysis. All statistical tests were two-sided. A *p* value < 0.05 was considered statistically significant. Statistical significance is indicated as follows: *p* ≤ 0.05 (*), *p* ≤ 0.01 (**), and *p* ≤ 0.001 (***).

## 5. Conclusions

This study demonstrates that purified Gram-positive PG exerts local antitumor immunomodulatory activity in a murine B16-F10 melanoma model under the tested conditions. Although PG reproduced several functional features observed with the insoluble *C. acnes* fraction, the present study does not establish PG as the component responsible for the antitumor activity of *C. acnes*. These findings support further investigation of PG-based local immunomodulation in melanoma.

## Figures and Tables

**Figure 1:**
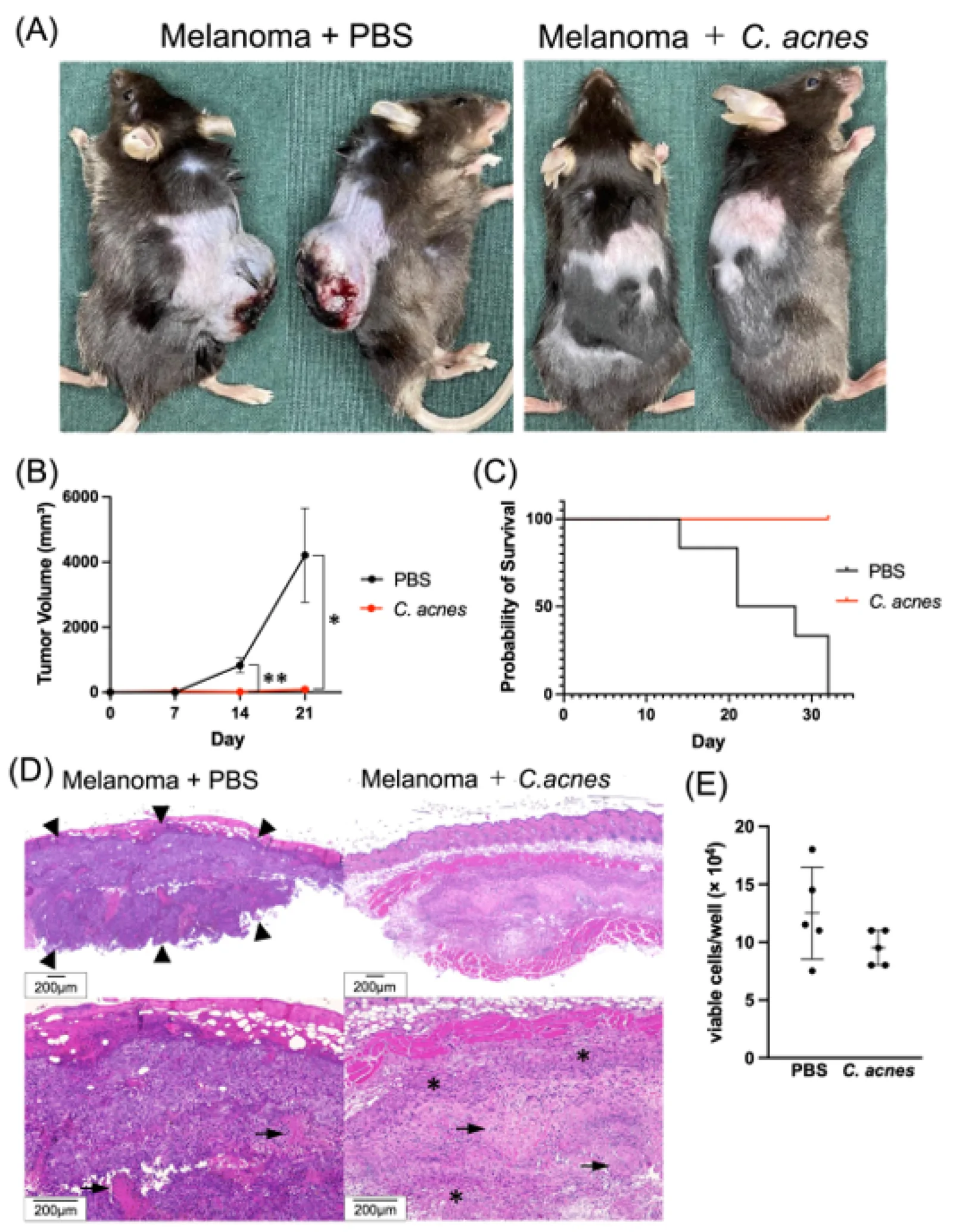
Reproduction of the antitumor activity of heat-killed *C. acnes* in a murine melanoma model. (**A**) Representative gross images of tumor-bearing mice at day 21 after melanoma inoculation. Mice receiving local co-administration of B16-F10 melanoma cells with heat-killed *Cutibacterium acnes* (*C. acnes*) exhibited marked suppression of subcutaneous tumor growth compared with PBS-treated controls. (**B**) Tumor growth curves. Mice were inoculated subcutaneously with melanoma cells (1.0 × 10^6^ cells/mouse) and received a subcutaneous co-injection of heat-killed *C. acnes* (3.3 mg/mouse; heat-treated at 60 °C for 60 min) or PBS at the tumor inoculation site on day 0, followed by a booster injection at the same site on day 7 without additional melanoma cells. Tumor volume was measured weekly. Data are presented as mean ± SEM (*n* = 6 per group). Statistical comparisons between groups at the indicated time points were performed using the Mann–Whitney U test; * *p* < 0.05, ** *p* < 0.01. (**C**) Kaplan–Meier survival curves. Survival was monitored for 32 days after tumor inoculation in a cohort separate from that used for tumor/tissue analysis (*n* = 5 per group). Statistical significance was evaluated using the log-rank (Mantel–Cox) test; *p* < 0.001. (**D**) Representative hematoxylin and eosin (H&E) staining of tumors harvested at day 21. PBS-treated tumors showed diffuse proliferation of viable tumor cells with minimal necrosis, whereas heat-killed *C. acnes* treatment was associated with granulomatous inflammation and extensive tumor necrosis. Arrowheads indicate viable tumor areas, black arrows indicate necrotic foci, and asterisks indicate inflammatory cell infiltration/granulomatous changes. Scale bars, 200 μm. (**E**) Direct effect of heat-killed *C. acnes* on B16-F10 cells in vitro. B16-F10 cells were seeded at 1.0 × 10^4^ cells/well in 24-well plates and allowed to adhere overnight. Cells were subsequently treated with PBS or heat-killed *C. acnes* at 33 µg/well for 24 h. Both floating and adherent cells were collected, and viable cells were counted using the trypan blue exclusion method. Bars indicate the mean ± SD (*n* = 5 wells per condition). Statistical significance was assessed using the Mann–Whitney U test.

**Figure 2:**
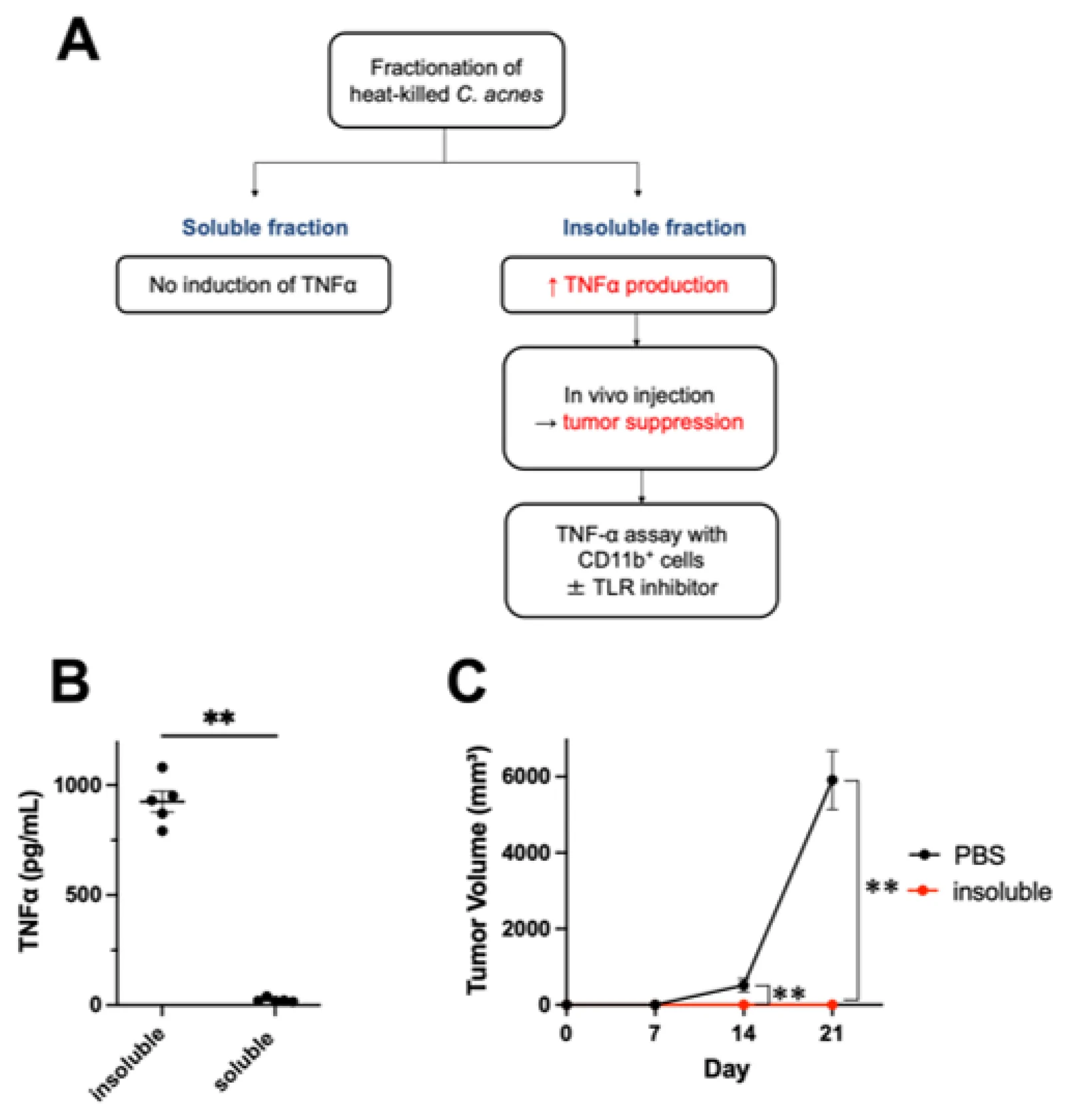
Functional dissection and activity profiling of *C. acnes*-derived fractions. (**A**) Experimental workflow for fractionation and downstream functional screening. After culturing and mechanically disrupting *C. acnes*, we separated bacterial components into soluble and insoluble fractions. The insoluble fraction showed greater TNF-α-inducing activity than the soluble fraction in total splenocytes and was subsequently evaluated in vivo. Black connecting arrows indicate the experimental workflow, and red arrows highlight the indicated responses. (**B**) TNF-α production by total splenocytes stimulated with soluble or insoluble *C. acnes* fractions. Splenocytes were stimulated for 3 h with the indicated fractions, and TNF-α in culture supernatants was quantified by ELISA. We added the insoluble fraction at 6.25 µg/well, and the soluble fraction at the same volume. Each dot represents an individual mouse (*n* = 5 per group). Statistical significance was assessed using the Mann–Whitney U test; ** *p* < 0.01. (**C**) Antitumor activity of the insoluble fraction in melanoma-bearing mice. On day 0, melanoma cells (1.0 × 10^6^ cells/mouse) were co-inoculated with the *C. acnes* insoluble fraction (3.3 mg/mouse), followed by re-administration of the same dose at the same site on day 7 without additional melanoma cells. The insoluble fraction significantly suppressed tumor growth compared with the control group. Data are shown as mean ± SEM (*n* = 5 per group). Group comparisons at the indicated time points were performed using the Mann–Whitney U test; ** *p* < 0.01.

**Figure 3:**
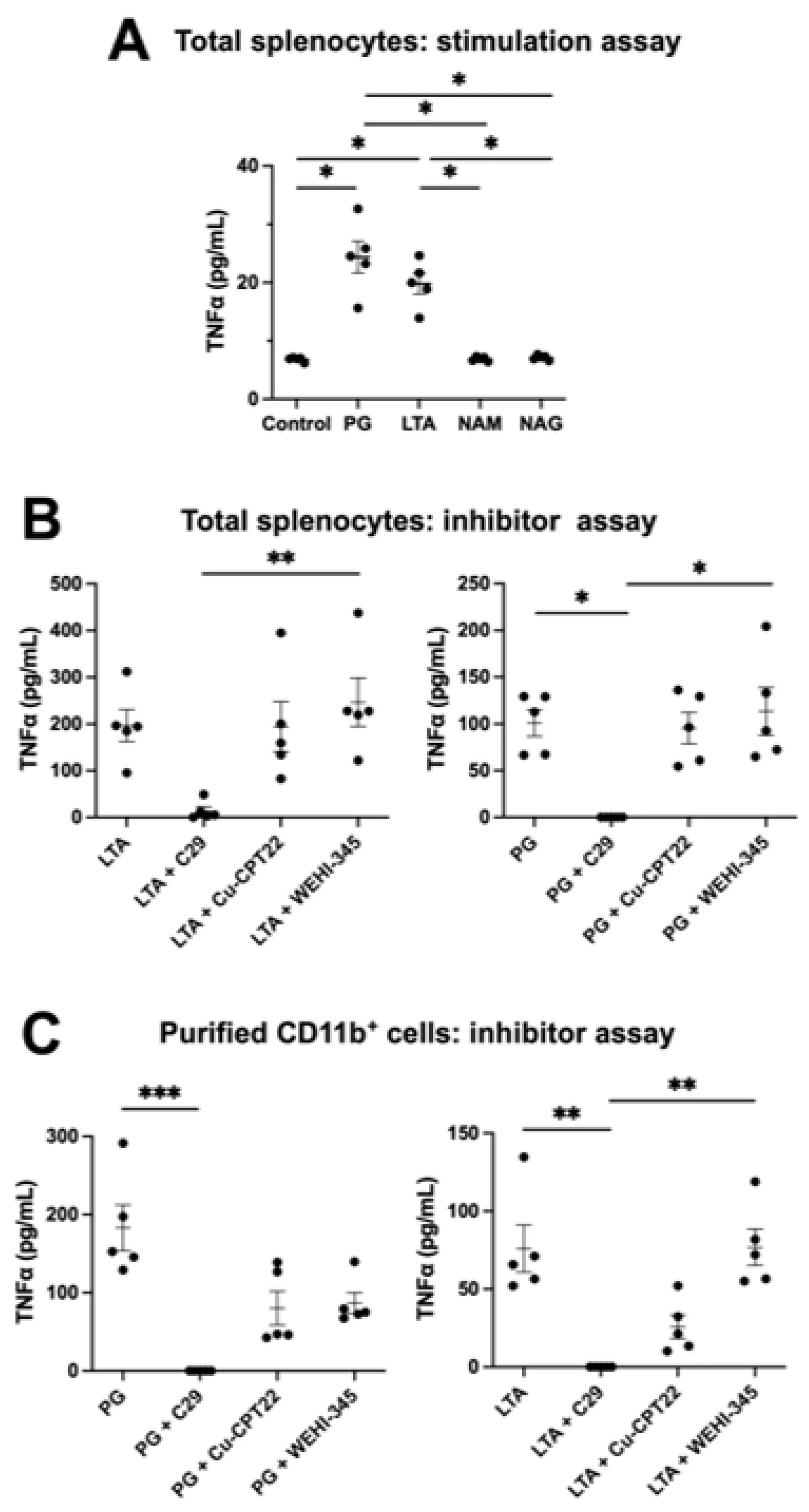
TNF-α induction by representative Gram-positive cell-wall components and pharmacologic assessment of TLR2 and RIPK2 pathway involvement. (**A**) TNF-α production in response to representative Gram-positive cell-wall components. Splenocytes were stimulated for 3 h with PG, LTA, N-acetylmuramic acid (NAM), or N-acetylglucosamine (NAG) (10 µg/well), and TNF-α in culture supernatants was quantified by ELISA. PG and LTA significantly increased TNF-α levels, whereas NAM and NAG did not. Data are shown as mean ± SEM; each dot represents an individual mouse (*n* = 5 per group). Statistics: one-way ANOVA followed by Tukey’s multiple-comparisons test; * *p* < 0.0001. (**B**) Effects of TLR2 and RIPK2 pathway inhibitors on PG- and LTA-induced TNF-α production in splenocytes. Splenocytes were pretreated for 3 h with the TLR2 inhibitors C29 or CU-CPT22, or the RIPK2 inhibitor WEHI-345, followed by stimulation with PG or LTA for 3 h. TNF-α in culture supernatants was measured by ELISA. Data are shown as mean ± SEM (*n* = 5 per group). Statistical significance was assessed using the Kruskal–Wallis test with multiple comparisons; * *p* < 0.05, ** *p* < 0.01. (**C**) Effects of TLR2 and RIPK2 pathway inhibitors on PG- and LTA-induced TNF-α production by purified CD11b^+^ cells. Splenic CD11b^+^ cells were pretreated for 3 h with the TLR2 inhibitors C29 or CU-CPT22, or the RIPK2 inhibitor WEHI-345, and then stimulated with PG or LTA for 3 h. TNF-α was quantified by ELISA. Data are shown as mean ± SEM (*n* = 5 per group). Statistical significance was assessed using the Kruskal–Wallis test with multiple comparisons; ** *p* < 0.01, *** *p* < 0.001.

**Figure 4:**
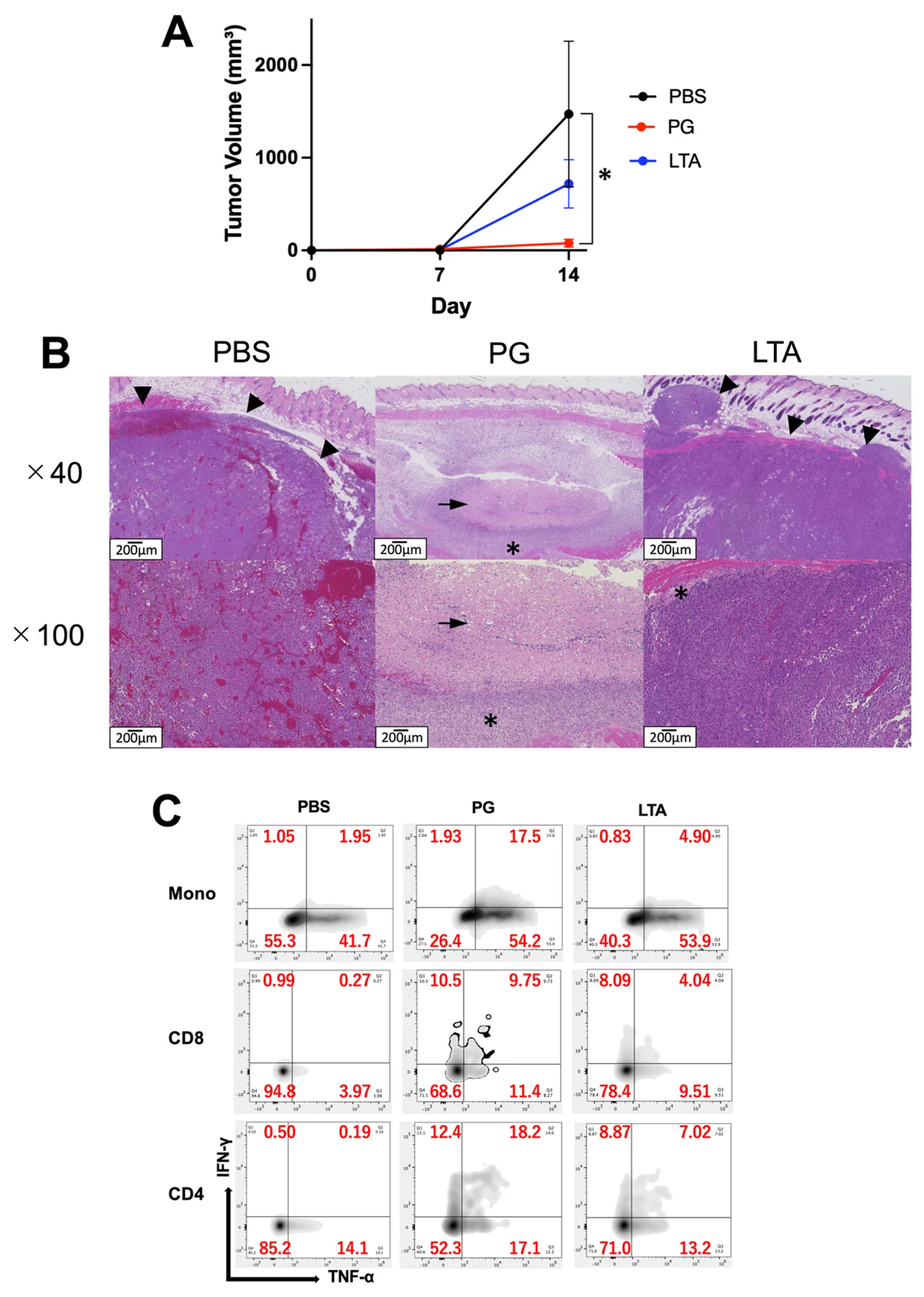
Comparative antitumor effects of PG and LTA. (**A**) Tumor growth kinetics in PBS-, PG-, and LTA-treated groups. C57BL/6NCrSlc mice were subcutaneously inoculated on day 0 with melanoma cells together with PBS, PG, or LTA. Then mice received an additional administration of PBS, PG, or LTA on day 7, and tumor volume was monitored longitudinally until day 14. The total administered doses were 2 mg/mouse for PG and 2.5 mg/mouse for LTA, divided equally between the day 0 and day 7 injections. PG markedly suppressed tumor growth compared with PBS and LTA. Data are shown as mean ± SEM (*n* = 5 per group). Statistical significance was assessed using the Kruskal–Wallis test; * *p* < 0.05. (**B**) Representative H&E-stained tumor sections at day 14. PBS-treated tumors showed diffuse proliferation of viable tumor cells. PG-treated tumors exhibited necrotic foci accompanied by granulomatous inflammation. LTA-treated tumors showed substantial residual tumor with prominent inflammatory cell infiltration. Arrowheads indicate viable tumor areas, black arrows indicate necrotic foci, and asterisks indicate inflammatory cell infiltration/granulomatous changes. Images are shown at ×40 and ×100 magnification. Scale bars, 200 μm. (**C**) Flow-cytometric assessment of tumor-infiltrating immune cells at day 14. Tumors were harvested from five mice per treatment group, and tumor-infiltrating cells were pooled within each group before staining and acquisition. Representative plots show TNF-α- and IFN-γ-producing cells in the indicated populations. PG treatment showed increased frequencies of cytokine-producing monocyte/myeloid cells, CD4^+^ T cells, and CD8^+^ T cells compared with PBS-treated controls, whereas LTA treatment showed less pronounced changes. Because samples were pooled within each treatment group, statistical comparisons were not performed for these flow cytometry data.

**Figure 5:**
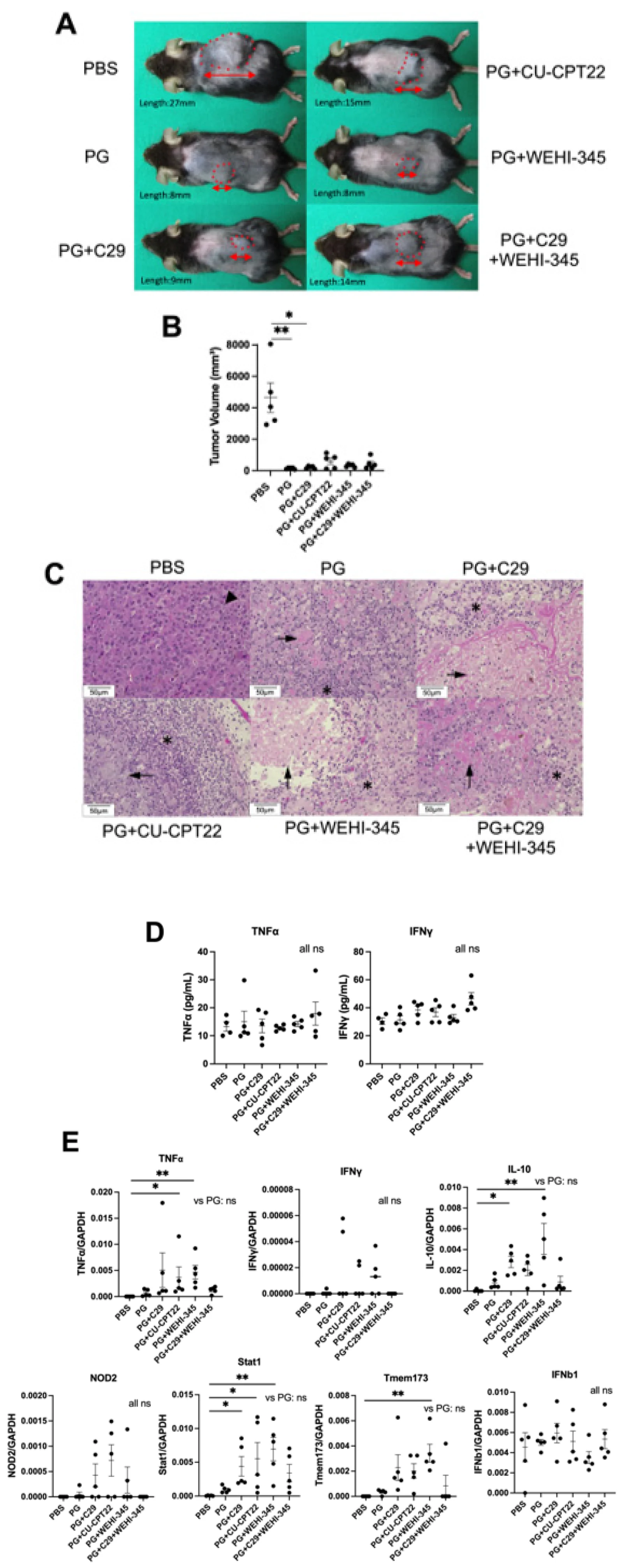
Pharmacologic assessment of TLR2 and RIPK2 pathway contributions to PG-associated antitumor activity. (**A**) Representative gross images of tumor-bearing WT C57BL/6NCrSlc mice. On day 0, melanoma cells were co-inoculated subcutaneously into the dorsal midline skin, slightly cranial to the pelvis, with PBS, PG, or PG combined with pathway inhibitors (C29, CU-CPT22, WEHI-345, or C29 + WEHI-345). PG was re-administered on day 7, and inhibitors were administered three times per week. Tumor growth was monitored until day 21 after tumor inoculation. Red lines indicate the approximate maximum diameter of the visible tumor mass. (**B**) Endpoint tumor volumes on day 21 are shown for PBS-treated controls, PG alone, and PG plus inhibitor groups. PBS was included as a baseline for tumor growth. The inhibitor-treated groups received PG together with C29, CU-CPT22, WEHI-345, or C29 plus WEHI-345. Each dot represents an individual mouse (*n* = 5 per group), and bars indicate mean ± SEM. Statistical analysis was performed using the Kruskal–Wallis test followed by Dunn’s multiple-comparisons test; significant differences are indicated in the panel. No inhibitor-treated group differed significantly from the PG-alone group in endpoint tumor volume. (**C**) Representative H&E-stained tumor sections harvested on day 21. PBS-treated tumors showed sheets of pleomorphic viable melanoma cells with minimal necrosis. In contrast, PG-treated tumors exhibited prominent inflammatory cell infiltration and extensive tumor necrosis. Similar tumor necrosis accompanied by inflammatory cell infiltration was observed in tumors from mice treated with PG in combination with TLR2 inhibitors (C29 or CU-CPT22) and/or the RIPK2 inhibitor WEHI-345. Arrowheads indicate viable tumor areas, black arrows indicate necrotic foci, and asterisks indicate inflammatory cell infiltration/granulomatous changes. Scale bar, 50 μm. (**D**) ELISA quantification of cytokines. TNF-α and IFN-γ levels were measured by ELISA in samples collected from mice treated with PBS, PG alone, or PG in combination with a TLR2 inhibitor (C29 or CU-CPT22) and/or a RIPK2 inhibitor (WEHI-345) on day 21. Each dot represents an individual mouse, and bars indicate mean ± SEM. Statistical analysis was performed using the Kruskal–Wallis test. “all ns” indicates no statistically significant differences among the groups tested. (**E**) qRT-PCR analysis of inflammatory and innate immune genes in tumors from the Figure 5A–C experiment. Total RNA was extracted from tumors, and the expression of *Tnf* (TNFα), *Ifng* (IFNγ), *Il10* (IL-10), *Stat1*, *Nod2*, *Tmem173* (STING), and *Ifnb1* (IFN-β) was quantified by qRT-PCR. Transcript levels were normalized to *Gapdh* and are shown as gene/GAPDH ratios. Each dot represents an individual mouse; bars indicate mean ± SEM. Statistical analysis was performed using the Kruskal–Wallis test with Dunn’s multiple-comparisons test. Significant differences are indicated in the panels (* *p* < 0.05, ** *p* < 0.01). “vs. PG: ns” indicates no statistically significant difference compared with the PG-alone group, and “all ns” indicates no statistically significant differences among the groups tested.

## Data Availability

The data presented in this study are available from the corresponding author upon reasonable request.

## Abbreviations

The following abbreviations are used in this manuscript:

ACK	ammonium–chloride–potassium
DAMP	damage-associated molecular pattern
DMSO	dimethyl sulfoxide
ELISA	enzyme-linked immunosorbent assay
H&E	hematoxylin and eosin
IFN	interferon
IL	interleukin
LTA	lipoteichoic acid
MACS	magnetic-activated cell sorting
NAG	N-acetyl-D-glucosamine
NAM	N-acetylmuramic acid
NF-κB	nuclear factor kappa B
NOD2	nucleotide-binding oligomerization domain-containing protein 2
PBS	phosphate-buffered saline
PG	peptidoglycan
PMA	phorbol 12-myristate 13-acetate
PGRP	peptidoglycan recognition protein
RIPK2	receptor-interacting serine/threonine-protein kinase 2
SEM	standard error of the mean
STING	stimulator of interferon genes
TLR2	Toll-like receptor 2
TNF	tumor necrosis factor
WT	wild-type
